# Capabilities of Cephalometric Methods to Study X-rays in Three-Dimensional Space (Review)

**DOI:** 10.17691/stm2024.16.3.07

**Published:** 2024-06-28

**Authors:** I.O. Ayupova, A.Yu. Makhota, A.V. Kolsanov, N.V. Popov, M.A. Davidyuk, I.A. Nekrasov, P.A. Romanova, A.M. Khamadeeva

**Affiliations:** MD, PhD, Associate Professor, Department of Pediatric Dentistry and Orthodontics; Samara State Medical University, 89 Chapayevskaya St., Samara, 443099, Russia; Student, Institute of Dentistry; Samara State Medical University, 89 Chapayevskaya St., Samara, 443099, Russia; MD, DSc, Professor of the Russian Academy of Sciences, Head of the Department of Operative Surgery and Clinical Anatomy with Innovation Technology Course; Samara State Medical University, 89 Chapayevskaya St., Samara, 443099, Russia Rector; Samara State Medical University, 89 Chapayevskaya St., Samara, 443099, Russia; MD, DSc, Associate Professor, Department of Pediatric Dentistry and Orthodontics; Samara State Medical University, 89 Chapayevskaya St., Samara, 443099, Russia; Bachelor of Computer Science; University of the People, 595 E. Colorado Boulevard, Suite 623, Pasadena, California, 91101, USA; Student, Faculty of Dentistry; The Patrice Lumumba Peoples’ Friendship University of Russia, 6 Miklukho-Maklaya St., Moscow, 117198, Russia; Student, Faculty of Dentistry; Tver State Medical University, 4 Sovetskaya St., Tver, 170100, Russia; MD, DSc, Professor, Department of Pediatric Dentistry and Orthodontics; Samara State Medical University, 89 Chapayevskaya St., Samara, 443099, Russia

**Keywords:** three-dimensional cephalometry, three-dimensional cephalometric analysis, orthodontics, asymmetric MFA deformities, maxillofacial anomalies, 3D cephalometry, CBCT

## Abstract

**The aim of the study** was a systematic review of modern methods of three-dimensional cephalometric analysis, and the assessment of their efficiency.

The scientific papers describing modern diagnostic methods of MFA in dental practice were searched in databases PubMed, Web of Science, eLIBRARY.RU, as well as in a searching system Google Scholar by the following key words: three-dimensional cephalometry, three-dimensional cephalometric analysis, orthodontics, asymmetric deformities, maxillofacial anomalies, 3D cephalometry, CBCT.

The literature analysis showed many methods of cephalometric analysis described as three-dimensional to use two-dimensional reformates for measurements. True three-dimensional methods are not applicable for practical purposes due to the fragmentary nature of the studies. There is the disunity in choosing landmarks and supporting planes that makes the diagnosis difficult and costly. The major issue is the lack of uniform standards for tree-dimensional measurements of anatomical structures of the skull, and the data revealed can be compared to them. In this regard, the use of artificial neuron networks and in-depth study technologies to process three-dimensional images and determining standard indicators appear to be promising.

## Introduction

Orthodontics as well as orthopedic and surgical dentistry requires thorough examination of patients’ maxillofacial area (MFA) to achieve the balance of facial structures [1–3]. For this purpose there can be used a cephalometric analysis of X-rays, biometric measurements of diagnostic gnathic models, a photo protocol, and other techniques [4–9]. The integrated use of methods determining unique anthropometric parameters enables to obtain a full-sized image of the patient’s MFA pathological condition [10–13]. Due to high informativity the preference is given to various X-ray examinations, since they have the methods for data acquisition and measurements.

It is well-known that using two-dimensional X-rays alone is inadequate to obtain diagnostic data [14–21], and the errors of two-dimensional X-rays [22] have motivated their quality improvement [23] and initiated the search for new kinds of examination.

As early as from the late XX century, cone-beam computed tomography (CBCT) has been used in practical medicine. As it advanced, it became safe for patients and more informative for specialists [24–30]. three-dimensional images are characterized by their detailed characteristics, it enables to assess MFA symmetry due to no image distortions of the bone structure outlines, and plot the coordinates of some anatomic landmarks located deeply in the skull space [16, 23, 31–36]. One of the main advantages of cephalometric analysis in three-dimensional space is the accuracy increase in identifying anatomic landmarks necessary to calculate anthropometric parameters [37–40].

In recent years, an increasing number of studies in dentistry aim at developing craniometric diagnostic methods in three-dimensional space using CBCT data [41–45]. All the above mentioned determined the relevance of the present study.

**The aim of the study** was to do a review of cephalometric methods of three-dimensional analysis, and the assessment of their efficiency.

## Materials and Methods

Publications were searched in scientific information systems PubMed, Google Scholar, Web of Science, and eLIBRARY.RU by the following key words: three-dimensional cephalometry, three-dimensional cephalometric analysis, orthodontics, asymmetric MFA deformities, maxillofacial anomalies, 3D cephalometry, CBCT.

The articles were chosen by two experts independently of one another, and all differences in opinions are smoothes on the basis of consensus, as well as through consultations with the third expert. The material relevance was assessed by the criteria of purpose fitting and the completeness of information using Statistica 10.0.1011. The repeated studies were eliminated, while those having no access to a full-text version were not considered. Key word searching in October 2023 revealed 9842 publications, and among them 94 completely corresponded to the topic and the research line.

## Results and Discussion

The literature review stated some authors to have described their own techniques of three-dimensional cephalmetric analysis; however, all measurements were made on image reformates in two-coordinate system, while in three planes only landmarks were determined in order to improve the quality of landmarks identification.

Treil et al. (1999) [46] developed the technique based on CBCT data to calculate the location of anatomic landmarks, where the image reformate was in relation to the sagittal plane following by calculating the angle and linear parameters in the two-coordinate system. The method suggested by the authors enabled to determine the incisor inclination and face height, though it disregarded other peculiarities of patient’s MFA. In addition, they failed to describe the landmarks, the authors based upon when constricting the sagittal plane, so it can cause disagreements in obtaining data.

**Figure 1.**
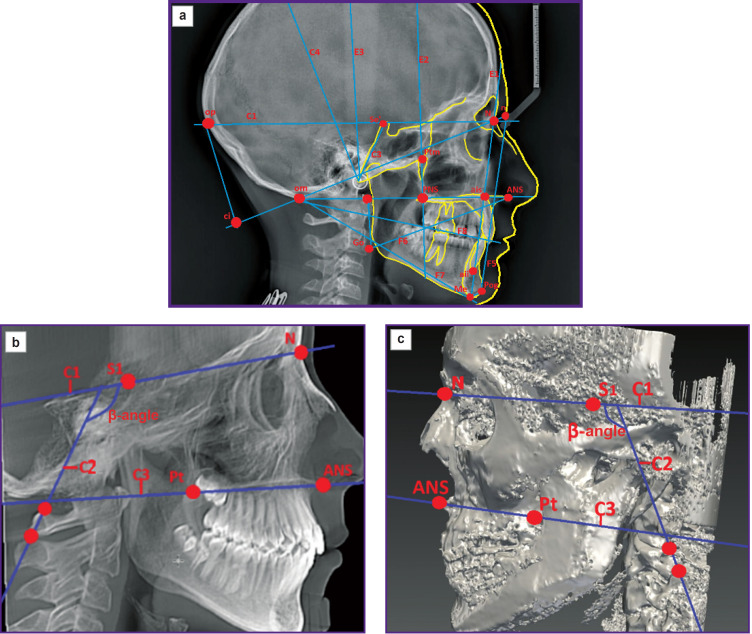
Three-dimensional cephalometric analysis according to Olszewski et al. [47]: (a) imaging planes: C1–C4; E1–E3; F5–F8; (b) imaging planes: С1 — the line connecting points S1 and N; С2 — the line drawn through the apexes of C3 and C4 spinous processes to C1 plane; C3 — the line drawn through the points ANS and Pt to C3 plane; (c) imaging planes: С1 — the line connecting points S1 and N; С2 — the line drawn through the apexes of C2 and C3 spinous processes to C1 plane; C3 — the line drawn through the points ANS and Pt to C3 plane

Many methods using image reformates for measurements have difficulties in constructing the planes for image orientation. So, Olszewski et al. (2006) [47] developed the method of three-dimensional tomographic cephalometry to identify the landmarks suggesting for these purpose 3 cranial (C1–C3) and 9 craniofacial planes (F1–F8, MP) (Figure 1). However, all calculations were made on skull reconstructions in relation to the sagittal plane, which was constructed by maxillary points, so it was impossible to conclude on mandibular bone structures symmetry [47], since its displacement from the plane can be caused by joint rotation [48].

Other methods have no clearly defined methodology of reformate elaboration. So, Swennen and Schutyser (2006) [49] tried to improve craniometric analysis using new landmarks, in relation to which it was possible to determine the sizes of anatomical structures, previously visually unavailable on two-dimensional images due to their deep location in the skull. The method advantage was proved by a comparative analysis of teleroentgenogram (TRG) and CBCT data, though there was no explanation how to construct the sagittal plane.

In 2022 Baldini et al. [50] carried out a comparative analysis of TRG data and CBCT reconstructions of the left and right MFA sides of one and the same patient. The measurements were taken according to a classical Steiner method including work with 8 linear and 7 angle parameters determined in an automated mode in vertical, sagittal and transverse directions. The measurements included the length calculations of the segments: PNS–A; S–N; N–Me; N–ANS; ANS–Me; Go– Me; Go–S; Go–Co; SNA, SNB, ANB; BaSN; S–N^PNS–ANS; PNS–ANS^Go–Me; S–N^Go–Me with constructing the midsagittal plane passing through the points Ba, Se, and N; axial — M, S, MSP; as well as coronary, which is perpendicular to the axial plane. Based on the study findings, there were no significant differences found when measuring the parameters based on TRG and CBCT data. Previously, other researchers [51] in the study devoted to the mandibular body measurements on the right and left, revealed the variability of point Me position. It emphasizes the significance of determining bone structures in three-dimensional space to understand clinical setting of a particular patient. The difference of opinion can be caused by the fact that Baldini et al. [50] excluded from the study CBCT of patients with asymmetric MFA deformities, and used simplified algorithms for calculating anthropometric parameters, as well as constructing the sagittal plane without regard to the mandibular defects.

Thus, the above mentioned techniques are not the methods to be used to determine parameters in true three-dimensional space, since all measurements are taken on two-dimensional surface. Moreover, fragmentary data on methods do not provide a complex presentation on MFA structure. Frequently, the represented findings are exclusively based on authors’ subjective evaluation, not resulting from statistical calculation and clinical efficiency. Many sources [46–50] have no information on the methods used to measure anthropometrical parameters in true three-dimensional space providing no data on developed standards for the parameters of anatomical landmarks relating to reference planes. The authors also base on the existing methods of two-dimensional studies giving no detailed description of the plane relating to which lateral cephalograms are reconstructed.

It is reasonable to make use of CBCT data preserving true orientation of some anatomical landmarks in three-dimensional space to be able to plan orthodontic [52] or orthognathic treatment [53–55] (especially in patients with asymmetric MFA deformities [56], as well as in temporomandibular joint (TMJ) dysfunction [57]), dental arch measuring [58] and when determining bone tissue volume of the alveolar process in implantation [59, 60].

**Figure 2.**
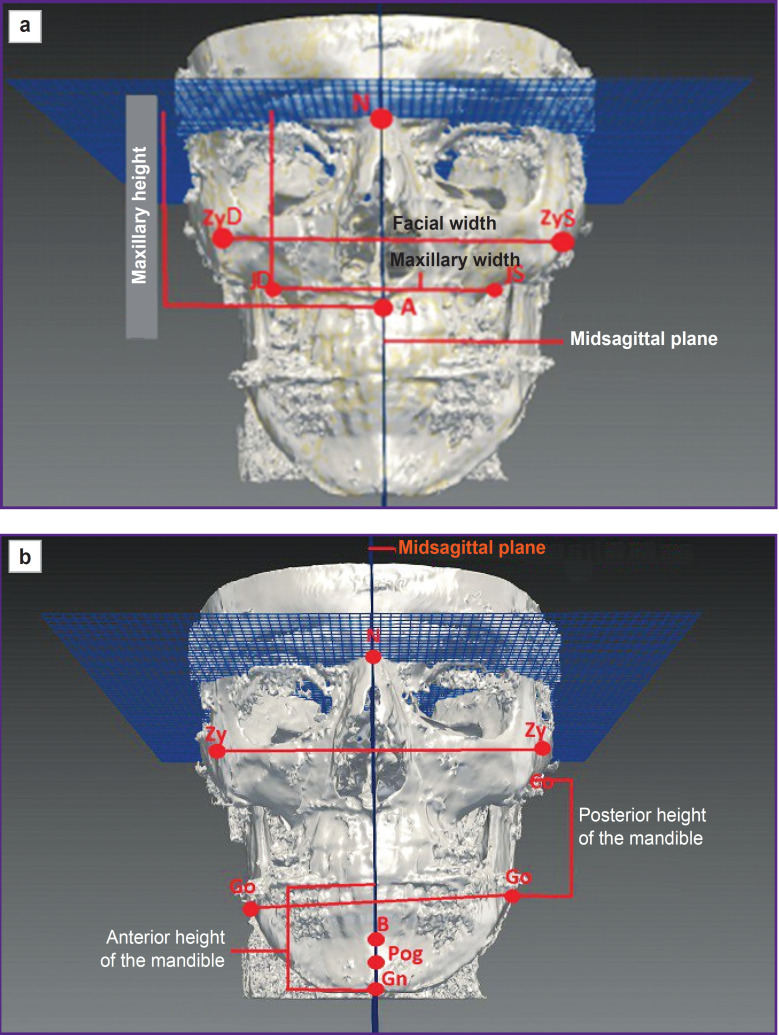
Three-dimensional craniometry according to Jacobson and Jacobson [61]: (a) midsagittal plane through N and A points; determining the maxillary height (A–N); the maxillary width (JD–JS); facial width (ZyD, ZyS); (b) determining the posterior height of the mandible (Pt–Go); the anterior height of the mandible (OP–Gn)

The attempts to develop and improve three-dimensional craniometry have been frequently made by researchers. As early as in 1994 Jacobson and Jacobson [61] suggested the method consisting in combining CBCT data to calculate hard tissue parameters and a facial scan to obtain the information on soft tissue condition. They attempted to determine the anthropometric norms, in relation to which it would be possible to measure cephalometric parameters in true three-dimensional space. The analysis was made using a program complex for cephalometry. On the skull base there were successively placed 29 basis points necessary for measurements. As reference planes there were used the following ones: the anterior facial plane — as a reference plane to assess the true position of the nose, lips and the chin in relation to point А; the superior and inferior, the right and left facial lateral, as well as the middle sagittal planes (Figure 2). In measurements they managed to determine true position of the maxilla in harmonically developed face; the position of soft nasal tissues, upper and lower lips, and the chin in relation to the anterior facial plane; the mandibular position in relation to the anterior skull base.

The advantage of the suggested method [61] consists in clearly defined, though singular, MFA standard parameters (total face height, its upper and lower parts, the vertical sizes of the chin and facial width for males and females, as well as for certain skull forms depending on patient’s ethnicity). The method value decreases the absence of attention to the mandibular sagittal plane as a separate mobile skull structure. Despite the measurability of angular and linear parameters of a three-dimensional image and the determination of the distance between anthropometric points deeply located in anatomical skull spaces, the essential fault is no data on significant verified anthropometric standards of skull parameters.

The more informative method was suggested by Bettega et al. (2000) [62]. To determine the true maxillary position in relation to the skull base they made a cephalometric analysis based on Delarie technique. 12 anatomical landmarks were used to study MFA: symmetric (Me, Pcp, fm) and non-symmetric (N, ANS, PNS, np, chin, chin’), as well as 2 angles: α and β. Using these anatomical landmarks they constructed reference planes and made a cephalometric analysis. During the investigation the authors succeeded in determining maxillary and mandibular re- and progenia, and identifying their position related to the skull base. The shortcoming of the suggested method is the midsagittal plane constructing through the points located on the skull base of the maxilla and mandible, i.e. the approach does not consider possible asymmetric MFA deformities, when these anatomical landmarks can be located in different planes. It can result in severe distortions in findings.

**Figure 3.**
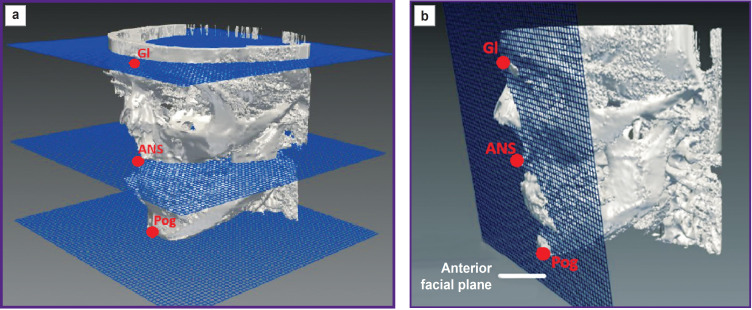
Three-dimensional cephalometric analysis using Total Face Approach [63, 64]: (a) the axial plane through the points Gl (frontal plane), ANS (anterior nasal spine plane), Pog (chin plane); (b) the anterior facial plane through the points Gl, ANS, Pog

One of the attempts to create a true three-dimensional cephalometry was a complex analysis: Total Face Approach (TFA) suggested by Perrotti et al. (2017) [63, 64]. The method was the algorithm used to determine the symmetry of facial structures, sagittal correlations of the jaws and the site of some anthropometric landmarks, and enabled to classify the growth type of facial structures based on CBCT. The analysis purpose was to determine the true size of anatomical structures without deviation of the parameters typical for two-dimensional images. During the study, they made calculations on 36 CBCT in three mutually perpendicular planes. In zero position, check-points were placed, and in relation to the points there were further measured angular and linear dimensions of anatomical skull structures by determining the distance between the initial point and the reference plane. TFA consisted in constructing 4 planes in an axial view, which were parallel to each other: the anterior facial plane, the frontal nasal spine plane, the genian plane, as well as the frontal plane (Figure 3). The distances between the planes were measured with respect to one point and one plane: e.g., anterior superior vertical dimensions determined the distance between SFP (superior facial plane) and ANS (anterior nasal spine) and the distance between ANSP (anterior nasal spine plane) and Me; the full anterior vertical sizes — the distance between MP (mental plane) and Nasion.

The advantage of TFA system was in additional use of MSCT (multispiral computed tomography) data [23, 65, 66]. It enabled to get a detailed insight about soft and hard tissues of the MFA improving the accuracy of cephalometric examination [67].

Retrospective study analysis and small CBCT sample volume prevent from the overall estimating TFA efficiency. Theoretical application of the approach could contribute to the development of standardized norms of anthropometric parameters necessary for cephalometric analysis in true three-dimensional space. However, current lack of such norms places in question TFA feasibility for full-size study of MFA structures [65–67].

Three-dimensional skull images are also highly sought in orthodontics and oral surgery to determine the position of certain teeth [68–70].

CBCT findings enable to obtain the data on impacted teeth in an the alveolar process, and manipulate with images improving the identification accuracy of dental position [71–74].

A group of Russian researchers (2018) [75] suggested the technique to determine the bedding degree of impacted teeth, as well as the angular axis of impacted teeth in the frontal part of the maxillary bases. In order to get the data on incisors and canines topography on CBCT-images in the sagittal plane, they marked SNA and SNP points followed by connecting the points by a segment SNA–SNP. Then, on the apical maxillary basis they constructed the plane, and the vertical distance between its segments was subdivided into 3 equal parts, horizontal lines drawn through each point. When crossing the resulting lines there formed the angles, in relation to which it was possible to determine the true position of impacted teeth, and assess the difficulty in placing a tooth in a tooth row in the course of orthodontic treatment depending on the degree the tooth embedded in the alveolar process. The method supposed to determine an inclination axis of an impacted tooth by measuring the angle, one side of which is the middle axis of the impacted tooth. The disadvantages of the method are the limited range of the parameters to determine and the positioning the impacted teeth of the frontal area exclusively with no possibility to study the localization of premolars and molars.

**Figure 4.**
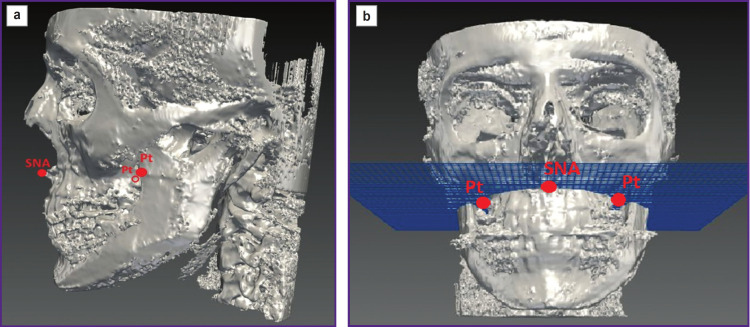
Determining the maxillary position according to Arkhipova and Arkhipov [76]: (a) the analysis of cone beam computed tomography image in the lateral view by constricting the plane going through the points SNA, Pt on the right and left; (b) the analysis of cone beam computed tomography image in the frontal view by constructing the plane going through the points SNA, Pt on the right and left

Another group of Russian researchers made efforts to work with X-rays in three-dimensional space. So, in 2019 there was suggested the technique of determining the true position of the maxilla in three-dimensional space [76]. The patient’s CBCT findings were processed in the software for converting them into two-dimensional cephalograms. On the obtained image sections there was marked ANS point, and Pt–M points on the right and left, and through these points the palatine plane was constructed (ANS–Pt–M-plane), it corresponding to the maxillary true position (Figure 4). The shortcoming of the suggested technique consisted in the inability to obtain numeric values to determine the maxillary position, as well as to measure the distance from the structures under study to other skull structures. Moreover, the method cannot be applied to the mandible.

Maspero et al. (2020) [77] suggested the method to determine the mandibular size and its growth direction. On the mandibular body there were successively marked the points GoR (Go Right), GoL (Go Left) and Me, and calculated angular and linear parameters between the resulting segments. The authors proved the necessity to determine the points of bone structures in three-dimensional space for thorough understanding a clinical situation of a certain patient.

**Figure 5.**
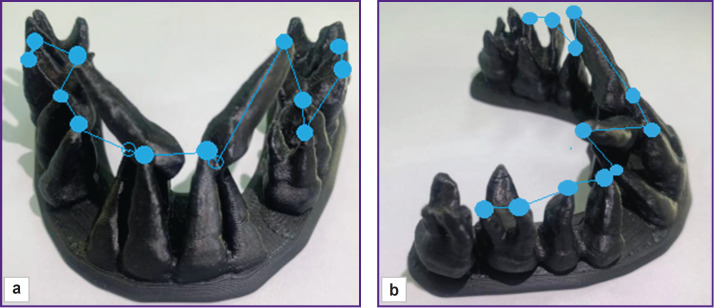
Dentition model to measure the apical maxillary basis according to Ishchenko and Popov [78]

To assess the apical basis of the maxilla, Ishchenko and Popov (2022) [78] performed CBCT, and converted the findings into STL-format to model teeth (Figure 5). When modeling, they removed the voxels representing the bone tissue of the alveolar process; thereby it was possible to make measurements in relation to dental roots. In multi-rooted teeth the measurements were made by buccal roots terminating the disto-buccal root of the permanent maxillary molar and the distal mandibular root. To interpret the findings the authors determined the ratio norms of mesio-distal sizes of 12 upper teeth to the length and width of the apical basis in certain dental groups. The right and the left jaw sides were studied separately to reveal the asymmetry degree and comparing to the apical basis width measured as the distance between distal roots of the first premolars and disto-buccal roots of the first permanent molars on either side. Then, comparing the findings with mesiodistal crown dimensions of 12 permanent teeth, they determined the presence of the necessary space for teeth from the left or right jaw sides. The authors suggest determining mesio-distal sizes from the contact dental points, since it can give an idea on the dentition extension; however, the method fails to calculate the site necessary for placing the existing teeth. The apical jaw basis parameters are impossible to measure by dental root apices, since in anomalies in the vertical and vestibulo-oral directions the results can be distorted, and it can result in lengthening of the measured segments. An essential fault of the technique is inability to determine the mandibular position in the skull space. In addition, the method is time- and labor-consuming, and requires special equipment.

**Figure 6.**
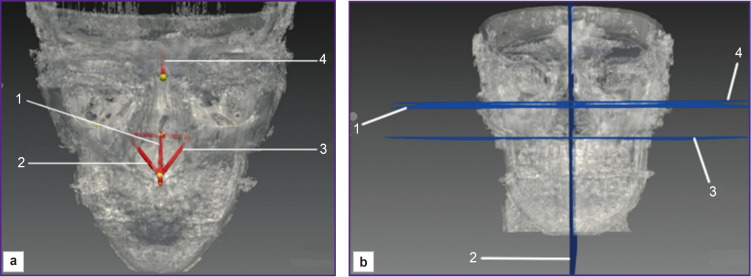
Determining true dimensions of the maxilla in three-dimensional space according to Kolsanov et al. [79]: (a) imaging segments: *1* — ANS–PNS; *2* — FPMID’ A’, maxillary length on the right; *3* — FPMIS’ A’, maxillary length on the left; *4* — NSe; (b) imaging planes: *1* — FHD (Frankfort plane dexter), the right Frankfurter horizontal plane; *2* — PSMax (planum saggitale maxillae), the maxillary sagittal plane; *3* — PN (planum nasale), the horizontal plane of the anterior and posterior nasal spines; *4* — FHS (Frankfort plane sinistra), the left Frankfurter horizontal plane

In order to determine true dimensions and position of the maxilla in relation to the anterior skull base in three-dimensional space, there was suggested the method [79] consisting in constructing Frankfurter plane, the midsagittal plane, the nasal plane and the anterior skull base plane using mathematical algorithms (Figure 6). The method enables to determine the maxilla true dimensions and position, and assess the symmetry degree of bone structures of both face halves in the research field; however, it provides no spatial relationship of the maxilla and the mandible.

The important task of three-dimensional cephalometric analysis is to determine the mandibular parameters. The mandibular body length measured by the distance between Ме and Go points is subject to significant projection distortions on TRG, it being proved by numerous studies [80–82].

**Figure 7.**
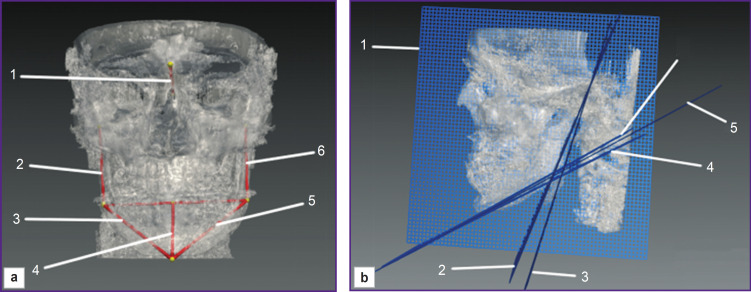
Determining true dimensions of the mandible in three-dimensional space using software [83]: (a) imaging segments: *1* — the segment connecting N and Se points; *2* — PRMD segment (plana ramus mandibulae dexter); *3* — PCMD segment (plana corpus mandibulae dexter); *4* — the perpendicular to the right and left planes of the mandibular rami; *5* — PCMS segment (plana corpus mandibulae sinister); *6* — PRMS segment (plana ramus mandibulae sinister); (b) imaging plane: *1* — PSM (plana sagittalia mandibula), mandibular sagittal plane; *2* — PRMS, the vertical plane of the left mandibular ramus; *3* — PRMD, the vertical plane of the right mandibular ramus; *4* — PCMS, the horizontal plane of the mandibular bode on the left; *5* — PMMI (plana media mandibulae inferioris), the horizontal plane of the mandibular body; *6* — PCMD, the horizontal plane of the mandibular body on the right

In 2023 a group of authors [83] devised the way enable to determine true mandibular size in threedimensional space using speciality applicationdependent software. So, it is possible to determine the length and symmetry degree of the mabdibular body and its rami by arranging anatomical landmarks in relation to certain planes (Figure 7); the authors suggested the sagittal plane, since it considers possible TMJ deviations, and enables to increase the diagnostic accuracy of bone structures of the right and left mandibular halves. The disadvantages of the method are the lack of the analysis of spatial gnathic relationship and data fragmentarity, it preventing from estimating other MFA structures and determining their growth direction.

The described research results [14, 79–83] enable to obtain the information on true dimensions and the position of the maxilla and the mandible with respect to other MFA structures, as well as determine their spatial symmetry. However, when making measurements, there were ignored other cephalometric parameters, and there were no values of anthropometric standards for anatomical structures.

Thus, there was proved the incompetence of current methods of three-dimensional cephalometric analysis, which consists in inconsistency and fragmentarity, as well as the lack of standardized norms, which can be compared with the measurements obtained.

## Conclusion

The literature analysis showed many methods of cephalometric analysis of X-rays described as threedimensional to be not actually three-dimensional, since they use two-dimensional reformates of images of patient’s maxillofacial area to measure angular and linear parameters. The methods using true three-dimensional analysis have contradictory and fragmentary information based on limited data. Moreover, there is the disunity in choosing landmarks and reference planes to measure required parameters. Some methods suggest combining two or more examinations that makes the diagnosis time-consuming and costly. Due to this reason they are unlikely to become widely used in clinical practice. Frequently, the fact that the mandible is a separate movable structure [84] requiring its own sagittal plane for correct diagnosis is overlooked. One of the main disadvantages of the existing methods is the lack of uniform standards for tree-dimensional measurements. Their development presupposes mass data analysis, and it is quite a task. Viewer programs in most cases have limited functions, it causing the necessity to develop methods to calculate the landmarks coordinates in space and determine their relationship, as well as add functionality of program complexes [85–88]. In this regard, the use of artificial neuron networks and in-depth study technologies to process three-dimensional images appear to be promising. These technologies can significantly facilitate the data collection to develop the standards, since they enable to determine the position of certain voxels reflecting some masses in the skull, and perform complex measurements [89–94].
